# Preattentive processing of emotional musical tones: a multidimensional scaling and ERP study

**DOI:** 10.3389/fpsyg.2013.00656

**Published:** 2013-09-23

**Authors:** Katja N. Spreckelmeyer, Eckart Altenmüller, Hans Colonius, Thomas F. Münte

**Affiliations:** ^1^Department of Psychology, Stanford UniversityStanford, CA, USA; ^2^Institute of Music Physiology and Musicians' Medicine, University of Music, Drama, and MediaHannover, Germany; ^3^Department of Psychology, University of OldenburgOldenburg, Germany; ^4^Department of Neurology, University of LübeckLübeck, Germany

**Keywords:** preattentive processing, musical emotion, timbre, event-related potential, mismatch negativity, multidimensional scaling

## Abstract

Musical emotion can be conveyed by subtle variations in timbre. Here, we investigated whether the brain is capable to discriminate tones differing in emotional expression by recording event-related potentials (ERPs) in an oddball paradigm under preattentive listening conditions. First, using multidimensional Fechnerian scaling, pairs of violin tones played with a happy or sad intonation were rated same or different by a group of non-musicians. Three happy and three sad tones were selected for the ERP experiment. The Fechnerian distances between tones within an emotion were in the same range as the distances between tones of different emotions. In two conditions, either 3 happy and 1 sad or 3 sad and 1 happy tone were presented in pseudo-random order. A mismatch negativity for the emotional deviant was observed, indicating that in spite of considerable perceptual differences between the three equiprobable tones of the standard emotion, a template was formed based on timbral cues against which the emotional deviant was compared. Based on Juslin's assumption of redundant code usage, we propose that tones were grouped together, because they were identified as belonging to one emotional category based on different emotion-specific cues. These results indicate that the brain forms an emotional memory trace at a preattentive level and thus, extends previous investigations in which emotional deviance was confounded with physical dissimilarity. Differences between sad and happy tones were observed which might be due to the fact that the happy emotion is mostly communicated by suprasegmental features.

## Introduction

Music, as well as language, can be used to transport emotional information and, from an evolutionary perspective, it does not come as a surprise that the way emotion is encoded in music is similar to the encoding of emotion in human or animal vocalizations. Interestingly, the emotional and semantic processing of speech has been shown to be supported by different brain systems by the method of double dissociation (e.g., Heilman et al., 1975). While six patients with right temporoparietal lesions and left unilateral neglect were demonstrated to have a deficit in the comprehension of affective speech, six patients with left temporoparietal lesions exhibited fluent aphasia, i.e., problems with the content of speech, but no problems with affective processing. Likewise, in music processing the Montreal group around Isabelle Peretz has described a patient that is selectively impaired in the deciphering of emotions from music while being unimpaired for the processing of other aspects of music (Peretz et al., 2001).

Researchers have tried to identify segmental and suprasegmental features that are used to encode emotional information in human speech, animal vocalizations, and music. With regard to animals, similar acoustic features are used by different species to communicate emotions (Owings and Morton, 1998). In humans, perceived emotion appears to be mainly driven by the mean level and the range of the fundamental frequency (F0) (Williams and Stevens, 1972; Scherer, 1988; Sloboda, 1990; Pihan et al., 2000) with low F0 being related to sadness and, conversely, high mean F0 level being related to happiness. In music, Hevner (1935, 1936, 1937) in her classical studies found that tempo and mode had the largest effects on listeners' judgments, followed by pitch level, harmony, and rhythm. According to Juslin (2001) musical features encoding sadness include slow mean tempo, legato articulation, small articulation variability, low sound level, dull timbre, large timing variations, soft duration contrasts, slow tone attacks, flat micro-intonation, slow vibrato, and final ritardando, whereas happiness is encoded by fast mean tempo, small tempo variability, staccato articulation, large articulation variability, fairly high sound level, little sound level variability, bright timbre, fast tone attacks, small timing variations, sharp duration contrasts, and rising micro-intonation.

While suprasegmental features are thought to be, at least in part, the result of a lifelong sociocultural conventionalization and therefore, maybe less hardwired (Sloboda, 1990), a considerable part of the emotional information is transmitted by segmental features concerning individual tones. For example, a single violin tone might be recognized as sad or happy with a rather high accuracy. Indeed, string and wind instruments which afford a high degree of control over the intonation can be used to mimic the segmental features also used by singers to convey emotional information.

Segmental emotional information can be encoded into a single tone by varying its timbre, which might be defined as reflecting the different quality of sounds aside from variations in pitch, loudness, and duration. In addition to different distributions of amplitudes of the harmonic components of a complex tone in a steady state (Helmholtz, 1885/1954), dynamic variations of the sound such as attack time and spectral flux (Grey, 1977; Grey and Moorer, 1977) are also important, particularly with regard to onset characteristics. Multidimensional scaling procedures on tones differing in timbre, because they were produced by different by different musical instruments, showed that this aspect of a tone is determined by variations along three dimensions termed attack time, spectral centroid, and spectral flux (McAdams et al., 1995). Likewise, in a recent study using multidimensional scaling (MDS) procedures to investigate the emotional information transmitted by variations in timbre, Eerola et al. (2012) found that affect dimensions could be explained in terms of three kinds of acoustic features: spectral (= ratio of high-frequency to low-frequency energy), temporal (= attack slope), and spectro-temporal (= spectral flux).

From the discussion above, there is no question as to the importance of detection of emotional timbre in voice and—by extension—in music. The question that we ask here pertains to *when* in the auditory processing stream emotional timbre is differentially processed. Given the high evolutionary benefit that might be afforded by the rapid decoding of emotional information from single tones (or human calls), we hypothesize that such information might be processed “early” in the processing stream and in an *automatic* fashion. Indeed, there are a number of studies that have investigated rapid and preattentive classification of emotional sounds. In particular, our group presented normal non-musician participants with tone series comprising a frequent (standard) single violin tone played with a certain emotional connotation (happy or sad) and a rare (deviant) violin tone played with the “opposite” intonation (Goydke et al., 2004). In parallel to the tone series, the EEG was recorded with a focus on the mismatch negativity (MMN). The MMN has been shown to be an ideal tool to address the early, automatic stages of sound evaluation (Näätänen, 1992; Picton et al., 2000; Näätänen et al., 2001). It is a component of the auditory event related potential (ERP) which is elicited during *passive* listening by an infrequent change in a repetitive series of sounds. In the original incarnation of the MMN paradigm, it occurs in response to any stimulus which is physically deviant (in frequency, duration or intensity) to the standard tone. Importantly, the standard stimulus in typical MMN experiments is the same throughout the experiment. It has been shown, however, that the MMN can also be obtained to deviations within complex series of sounds (Picton et al., 2000; Näätänen et al., 2001), in which the memory trace is defined by some abstract property (e.g., ascending series of tones). Thus, it appears that the notion of a standard/memory trace can be extended such that the auditory system is capable to extract systematic properties of sound series. Moreover, and important for Goydke et al. (2004) and the present study, the MMN is sensitive to changes in the spectral component of tonal timbre (Tervaniemi et al., 1997). The onset latency of the MMN varies according to the nature of the stimulus deviance. Whereas simple, physically deviant stimuli show an onset latency of the MMN of about 150 ms, much later MMNs have been seen with more complex forms of deviance. Finally, it is important to stress the fact that the analysis of the incoming stimulus as well as its encoding appears to take place automatically since the MMN typically occurs when the subjects do not attend to the eliciting stimuli, for example during engagement in a different task such as reading a book (Näätänen, 1992). Returning to the Goydke et al. (2004) study, deviant tones were associated with an MMN. The MMN scalp topography for the emotional deviant was similar to an MMN for a control pitch deviant tone. These results were taken to indicate that the brain can categorize tones preattentively on the basis of subtle cues related to the emotional status of the tone (Goydke et al., 2004). Studies using a similar logic using both emotionally voiced words (Schröder et al., 2006) or vocalizations (Bostanov and Kotchoubey, 2004) have revealed analogous findings. Further, investigating different timbral dimensions (attack time, spectral centroid, and spectrum fine structure) and their consequences for behavioral classification latencies and ERPs in preattentive (Caclin et al., 2006) and attentive (Caclin et al., 2008) listening conditions, Caclin and colleagues showed that these different timbral features are separately represented in sensory auditory memory.

One important aspect has been neglected by these studies, however, in the Goydke et al. (2004) study, a single (e.g., happy) tone was presented repeatedly as a standard and a single (e.g., sad) tone was presented repeatedly as the emotional deviant. Thus, it is possible, that the MMN observed for the deviants in this study might have been driven by the physical differences between the standard and deviant stimuli rather than by the postulated preattentive emotional categorization of the stimulus. Indeed, different mechanisms of deviance detection (termed sensory and cognitive) have been demonstrated for other types of stimulus materials (Schröger and Wolff, 1996; Jääskeläinen et al., 2004; Opitz et al., 2005).

Therefore, to answer this question and extend our previous findings (Goydke et al., 2004), we conducted the present study. As pointed out before, segmental features encoding emotion seem to be varied. Thus, what makes the study of acoustical emotion difficult is, that the set of features encoding the same emotion does not seem to be very well defined and that there is a great variance of feature combinations found within individual emotion categories. We modified the design of our previous MMN study to see whether affective expressions are pre-attentively categorized even when their acoustical structure differs. In other words, several (*n* = 3, probability of occurrence for each tone 25%) instances of sad (or happy) tones were defined as standards to which an equally probable deviant stimulus (25%) of the other emotion had to be compared preattentively. To the extent that the MMN reflects deviance in the sense of “being rare,” an MMN under these circumstances would indicate that the standards have been grouped to define a single “emotional” entity.

To test whether the brain automatically builds up categories of basic emotions across tones of different (psycho)-acoustical structure, it was necessary to create two sets of tones, where tones within one set could clearly be categorized as happy and sad, respectively but differed with respect to their acoustical structure. To this end, we first performed extensive studies to define the stimulus set for the MMN study using MDS methods. Two types of criteria were set for tones to be used as standards in the MMN study: first, each tone needed to be consistently categorized as happy or sad and, second, tones within one set as well as across sets needed to be perceived as different. The first point was addressed by performing affect-ratings on a set of violin tones which only differed in emotional expression but not in pitch or instrumental timbre. To tackle point 2, pairwise same-different-comparisons were collected for all tones and fed into a Fechnerian scaling procedure to assess the perceived similarity among the tones. We will first describe the scaling experiment and will then turn to the MMN experiment.

For the latter, we had a straightforward expectation: If the brain categorizes tones preattentively on the basis of an automatic emotional grouping, we should observe an MMN for emotional deviant stimuli regardless of the fact that these emotional deviants were as probable as each of the three different standard stimuli.

## Scaling experiment

Multidimensional Fechnerian scaling (Dzhafarov and Colonius, 1999, 2001) is a tool for studying the perceptual relationship among stimuli. The general aim of MDS is to arrange a set of stimuli in a low-dimensional (typically Euclidean) space such that the distances among the stimuli represent their subjective (dis)similarity as perceived by a group of judges. Judges generally perform their ratings in pairwise comparisons between all stimuli in question. Based on the dissimilarity data a MDS procedure finds the best fitting spatial constellation by use of a function minimization algorithm that evaluates different configurations with the goal of maximizing the goodness-of-fit (Kruskal, 1964a,b). Though the dimensions found to span the scaling space can often be interpreted as psychologically meaningful attributes that underlie the judgment, no a priori assumptions have to be made about the nature of the dimensions. Thus, with MDS perceptual similarity can be studied without the need to introduce predefined feature concepts (as labels for the dimensions) which might bias people's judgments.

Fechnerian scaling is a development of classical MDS which is more suitable to be used with psychophysical data. Dzhafarov and Colonius (2006) have pointed out that certain requirements for data to be used with classical MDS are usually violated in empirical data, namely the property of symmetry and the property of constant self-dissimilarity. The property of symmetry assumes that discrimination probability is independent of presentation order, and, thus, that the probability to judge a stimulus x as different from a stimulus y is the same no matter whether x or y is presented first [*p*(*x*; *y*) = *p*(*y*; *x*)]. It has been known since Fechner (1860) that this is not true. The property of constant self-dissimilarity expects that any given stimulus is never perceived as different from itself, thus, that the probability to judge stimulus x as different from itself is 0 [*p*(*x*; *x*) = *p*(*y*; *y*)]. However, it has been shown repeatedly that this is not the case in psychophysical data (e.g., Rothkopf, 1957). The only requirement made by Fechnerian scaling is that of regular minimality, requesting that the probability to judge a stimulus as different from itself needs to be lower than any other discrimination probability.

In the present experiment Fechnerian scaling is used to establish subjective distances for a set of tones where tones differ with respect to their emotional expression.

## Materials and methods

### Stimulus material

To generate the stimulus material, 9 female violinists (all students of the Hanover University for Music and Drama) were asked to play brief melodic phrases all ending on c-sharp. Melodies were to be played several times with happy, neutral, or sad expressions. Before each musician started with a new expression, she was shown a sequence of pictures from the IAPS (Lang et al., 2008) which depicted happy, neutral or sad scenes, to give her an idea of what was meant by happy, neutral, and sad. All violinists were recorded on the same day in the same room using the same recording technique: stereo (2 Neumann-microphones TLM127), 44.1 kHz sampling rate, 24 bit, distance from the instrument to the microphones was always 50 cm. Each musician filled out a form describing the changes in technique that she had applied to achieve the different expressions. From 200 melodic phrases the last tone (always c-sharp) was extracted using Adobe Audition. Only those tones were selected which were between 1450 and 1700 ms in length and had a pitch between 550 and 570 Hz. Tones from two violinists had to be discarded altogether because they were consistently below pitch level. The resulting pre-selection comprised 35 tones by 7 different violinists. To soften the tone onset a smooth fade-in envelope was created from 0 to 100 ms post-tone onset. The pre-selection was rated on a 5-point scale from very sad (1) to very happy (5) by 9 student subjects (mean age = 25.9 years, 5 males) naive to the purpose of the study and different from the participants taking part in the final experiment. Each tone was rated twice by each participant to test the raters' consistency. Tones were not amplitude-normalized, because it was found that differences in affective expression could not be differentiated properly in a normalized version. Based on the affect ratings and their consistency 10 tones were selected for the final stimulus set (Table 1).

**Table 1:** **Features of the stimulus material**.

**Tone**	**Duration (ms)**	**Frequency (Hz), (*SD*)**	**Mean level [dB(A)]**
tone01	1676	559.69 (2.41)	64.5
tone02	1526	558.99 (2.04)	66.2
tone03	1658	559.98 (4.45)	72.2
tone04	1628	554.39 (3.55)	71.6
tone05	1506	555.86 (1.13)	68.8
tone06	1534	561.86 (4.35)	68.5
tone07	1660	563.00 (4.58)	66.6
tone08	1630	561.31 (3.61)	67.8
tone09	1570	556.96 (1.25)	72.4
tone10	1608	557.64 (0.35)	68.8
Mean (*SD*)	1599 (61.5)	559.3 (2.75)	68.74 (2.66)

### Design of the same-different forced-choice experiment

Participants were 10 students (mean age = 25.4 years, 5 females) with no musical expertise who took part in two separate sessions. In session 1 they performed a same-different forced-choice task on the violin tones to provide data for MDS. In session 2 (approximately 1 week later) they were asked to rate the emotional expression of the tones on a five-point-scale.

For the forced-choice task, participants were tested individually while sitting in a comfortable chair 120 cm away from a 20-zoll-computer screen. All auditory stimuli were presented via closed head-phones (Beyerdynamic DT 770 M) with a level ranging from 64 to 73 dB. Presentation software (Neurobehavioral Systems) was used to present trials and to record responses. All 10 tones were combined with each other including themselves, resulting in 10 × 10 = 100 pairs; all 100 pairs were presented ten times, each time in a different randomized order (resulting in 1000 trials altogether). The stimulus onset asynchrony (SOA) between the two tones of a pair was 3500 ms. Participants had to strike one of two keys to respond same or different (forced choice). To make sure participants judged the psychoacoustical similarity of the tones unbiased, they were kept uninformed on the purpose of the experiment. Trial duration was about 6000 ms. The next trial was automatically started when one of the two buttons was pressed. Participants performed a short training to familiarize them with the procedure and were allowed to pause after each block of 25 trials. There were 40 blocks altogether. Participants could end the pause by pressing a button on the keyboard. The duration of the whole experiment was about 2 hours. Participants were verbally instructed to decide whether the two tones comprising a pair were same or different. For the data analysis responses were recorded as 0 (same) and 1 (different). Mean values (discrimination probabilities) per pair of tones were calculated over all participants and all responses. Minimum number of responses per pair was 90. The resulting discrimination probabilities were transformed into Fechnerian distances using FSDOS (Fechnerian Analysis of Discrete Object Sets by Dzhafarov and Colonius, see http://www.psych.purdue.edu/~ehtibar/).

### Affect rating

In session 2 each participant from the scaling experiment performed an affect rating of each individual violin tone. All stimuli were presented twice with the order being randomized for each participant. Participants were asked to rate each tone on a 5-point-scale ranging from very sad (1) to very happy (5) by pressing one of the keys from F1 to F5 on the keyboard. Emblematic faces illustrated the sad and the happy end of the scale.

### Valence and arousal rating

Stimulus material was also rated according to valence and arousal by two additional groups of participants. All stimuli were presented twice but the order was randomized for each participant. To give participants an idea what was meant by the terms valence and arousal they performed a short test trial on pictures taken from the IAPS. Group A (valence) (5 women, 5 men, mean age = 27.6) was asked to rate all 10 tones on a 5-point-scale ranging from very negative (1) to very positive (5). Group B (5 women, 5 men, mean age = 24.4) was asked to rate the 10 tones from very relaxed (German = “sehr entspannt”) (1) to highly aroused (German = “sehr erregt”) (5).

## Results

### Same-different forced-choice experiment

Discrimination probabilities for each pair of tones based on participants' same-different- judgments are shown in Table 2. Fechnerian distances for each pair of tones calculated from discrimination probabilities are shown in Table 3. Given values reflect the relative distances between pairs of tones as perceived by the mean participant. For example, tone04 (abbreviated t.04 in the row), is perceived about 1.5 times more distant from tone05 than from tone07.

**Table 2:** **Discrimination probabilities for the 10 tones**.

	**tone01**	**tone02**	**tone03**	**tone04**	**tone05**	**tone06**	**tone07**	**tone08**	**tone09**	**tone10**
t.01	0.06	0.12	1	0.89	0.74	0.81	0.86	0.94	0.88	0.89
t.02	0.16	0.08	0.98	0.91	0.69	0.72	0.85	0.89	0.88	0.93
t.03	0.99	0.97	0.04	0.93	0.97	0.93	0.85	0.88	0.98	0.95
t.04	0.9	0.93	0.96	0.08	0.82	0.42	0.51	0.64	0.6	0.96
t.05	0.7	0.77	1	0.84	0.08	0.79	0.85	0.91	0.78	0.74
t.06	0.89	0.8	0.94	0.62	0.93	0.07	0.3	0.35	0.74	0.79
t.07	0.92	0.91	0.97	0.69	0.86	0.41	0.09	0.2	0.89	0.93
t.08	0.9	0.91	0.94	0.75	0.9	0.31	0.16	0.1	0.86	0.83
t.09	0.88	0.95	0.96	0.66	0.82	0.77	0.8	0.76	0.08	0.26
t.10	0.91	0.94	1	0.91	0.65	0.77	0.89	0.82	0.34	0.06

Given are probabilities with which the mean perceiver judged the row tones to be different from the column tones.

**Table 3:** **Fechnerian distances**.

	**tone01**	**tone02**	**tone03**	**tone04**	**tone05**	**tone06**	**tone07**	**tone08**	**tone09**	**tone10**
t.01	0.000	0.140	1.890	1.650	1.290	1.510	1.630	1.670	1.620	1.680
t.02	0.140	0.000	1.830	1.680	1.290	1.370	1.590	1.620	1.660	1.730
t.03	1.890	1.830	0.000	1.770	1.850	1.760	1.690	1.680	1.820	1.850
t.04	1.650	1.680	1.770	0.000	1.500	0.890	1.030	1.190	1.100	1.550
t.05	1.290	1.290	1.850	1.500	0.000	1.570	1.540	1.630	1.440	1.250
t.06	1.510	1.370	1.760	0.890	1.570	0.000	0.550	0.490	1.360	1.430
t.07	1.630	1.590	1.690	1.030	1.540	0.550	0.000	0.170	1.520	1.660
t.08	1.670	1.620	1.680	1.190	1.630	0.490	0.170	0.000	1.440	1.490
t.09	1.620	1.660	1.820	1.100	1.440	1.360	1.520	1.440	0.000	0.460
t.10	1.680	1.730	1.850	1.550	1.250	1.430	1.660	1.490	0.460	0.000

Distances were calculated by FSDOS (the larger the value the more distant the tones).

### Affect, arousal, and valence rating

Results of the affect, arousal, and valence ratings are shown in Table 4 collapsed over the first and second presentation which did not differ significantly. Please note, that the affect rating was performed by the same group of participants that also took part in the same-different forced choice experiment, whereas the arousal and valence ratings were performed by two different groups of subjects. Though stemming from different groups of participants, there was a high correlation between the affect and the arousal ratings [*r* = 0.937, *p* < 0.001]. In contrast, the correlation between valence and affect ratings was rather low [*r* = 0.651, *p* = 0.042]. This is surprising for it was expected that valence and affect are closely related. It has to be noted, though, that during the testing it became apparent that participants used different concepts for the valence dimension. While some understood positive—negative in the sense of pleasant—unpleasant, others linked positive—negative to the two ends of the dimension to happy and sad. This problem is paralleled by a heterogeneous use of the valence-term in the literature (see Russell and Barrett, 1999, for a discussion) and might serve as an explanation for the incongruous pattern. In the current experiment the valence ratings will therefore, be interpreted with caution.

**Table 4:** **Results of the affect, arousal, and valence ratings**.

	**Affect**	**Arousal**	**Valence**	**Label**
tone01	1.90 (0.61)	1.75 (0.42)	2.80 (1.40)	sad01
tone02	1.95 (0.61)	1.90 (0.66)	3.20 (0.98)	sad02
tone03	4.40 (0.94)	4.55 (0.44)	3.55 (0.90)	
tone04	2.90 (0.39)	3.15 (1.00)	3.35 (0.67)	
tone05	2.20 (0.71)	1.80 (0.54)	2.70 (0.63)	sad03
tone06	2.70 (0.59)	3.00 (0.62)	3.25 (0.49)	
tone07	3.45 (0.98)	2.95 (0.55)	2.95 (0.44)	hap01
tone08	3.60 (0.77)	3.20 (0.71)	3.30 (0.63)	hap02
tone09	3.35 (0.71)	3.40 (0.81)	3.25 (1.03)	hap03
tone10	2.55 (0.55)	2.80 (0.63)	2.70 (1.01)	

Each scale ranged from 1 to 5; last column gives the label of the tone for the MMN study.

### Selection of stimuli for the MMN experiment

Three sad tones [tone01 (sad01), tone02 (sad02), tone05 (sad03)] and 3 happy tones [tone07 (hap01), tone08 (hap02), tone09 (hap03)] were chosen from the data set based on their affect ratings. The happy tones had mean affect ratings of 3.45, 3.60, and 3.35; sad tones were rated 1.90, 1.95, and 2.20, respectively. Affect ratings of happy and sad tones were significantly different [*F*_(9, 90)_ = 12.9 *p* < 0.001] and scaling procedures demonstrated that tones were perceived as different even when belonging to the same emotion category. Fechnerian distances between happy and sad tones fell between 1.44 and 1.67. Distances were 0.17, 1.52, and 1.44 among happy tones and 0.14 and 1.29 among sad tones.

## Event-related potential experiment

### Methods

#### Participants

Of a total of 19 participants three had to be excluded because of technical error (two) or too many blink artifacts in the ERP data (one). The remaining 16 participants (8 women) were aged between 21 and 29 years (mean = 24.9). None was a professional musician.

#### Design

Stimuli were the 6 different single violin tones chosen on the basis of the scaling experiment. Two conditions were set up in a modified oddball-design. In condition A 3 sad tones were presented in random order (standards) with 1 happy tone (deviant) randomly interspersed. In condition B 3 happy tones were presented as standards with 1 sad tone randomly interspersed as deviant tone. As deviants, the tones with the lowest and highest affect ratings were chosen. The probability of occurrence was 25% for each of the three standard tones and the deviant tone, resulting in an overall probability of 75% for the standard stimuli and 25% for the affective deviant. In both conditions each tone was presented 340 times resulting in a total of 1360 tones per condition. A randomization algorithm guaranteed that identical tones were never presented back-to-back. Both conditions were divided in two blocks of 680 tones. The order of blocks was ABAB or BABA. All four blocks were presented in one session with one pause between block 2 and 3. The total duration of the experiment was about 90 min.

Tones were presented via insert ear phones used with Earlink ear-tips (Aearo Comp.). Stimulus onset asynchrony between two tones was 2000 ms. Mean sound pressure level of the presentation of all tones was 70 dB. To realize a non-attentive listening paradigm, participants were instructed to pay attention to cartoons (Tom and Jerry—The classical collection 1) presented silently on a computer screen in front of them. To control how well participants had attended the film a difficult post-test was performed after the experiment requiring participants to recognize selected scenes. On average, 85% of the scenes were classified correctly, indicating that the participants had indeed attended the film.

#### ERP-recording

The electroencephalogram (EEG) was recorded from 32 tin electrodes mounted in an elastic cap according to the 10–20-system. Electrode impedance was kept below 5 kΩ. The EEG was amplified (bandpass 0.1–40 Hz) and digitized continuously at 250 Hz. Electrodes were referenced on-line to the left mastoid. Subsequently, off-line re-referencing to an electrode placed on the nose-tip was performed. Electrodes placed at the outer canthus of each eye were used to monitor horizontal eye movements. Vertical eye movements and blinks were monitored by electrodes above and below the right eye. Averages were obtained for 1024 ms epochs including a 100 ms pre-stimulus baseline period. Trials contaminated by eye movements or amplifier blocking or other artifacts within the critical time window were rejected prior to averaging. For this, different artifact rejection thresholds were defined for the eye- and EEG channels.

Separate averages were calculated for each tone in both conditions. ERPs were quantified by mean amplitude measures using the mean voltage of the 100 ms period preceding the onset of the stimulus as a reference. Time windows and electrode sites are specified at the appropriate places of the result section. Effects were tested for significance in separate ANOVAs, with stimulus type (standard or deviant) and electrode site as factors. The Huynh-Feldt epsilon correction (Huynh and Feldt, 1980) was used to correct for violations of the sphericity assumption. Reported are the original degrees of freedom and the corrected *p*-values. Significance level was set to *p* < 0.05.

### Results

**Figure 1:**
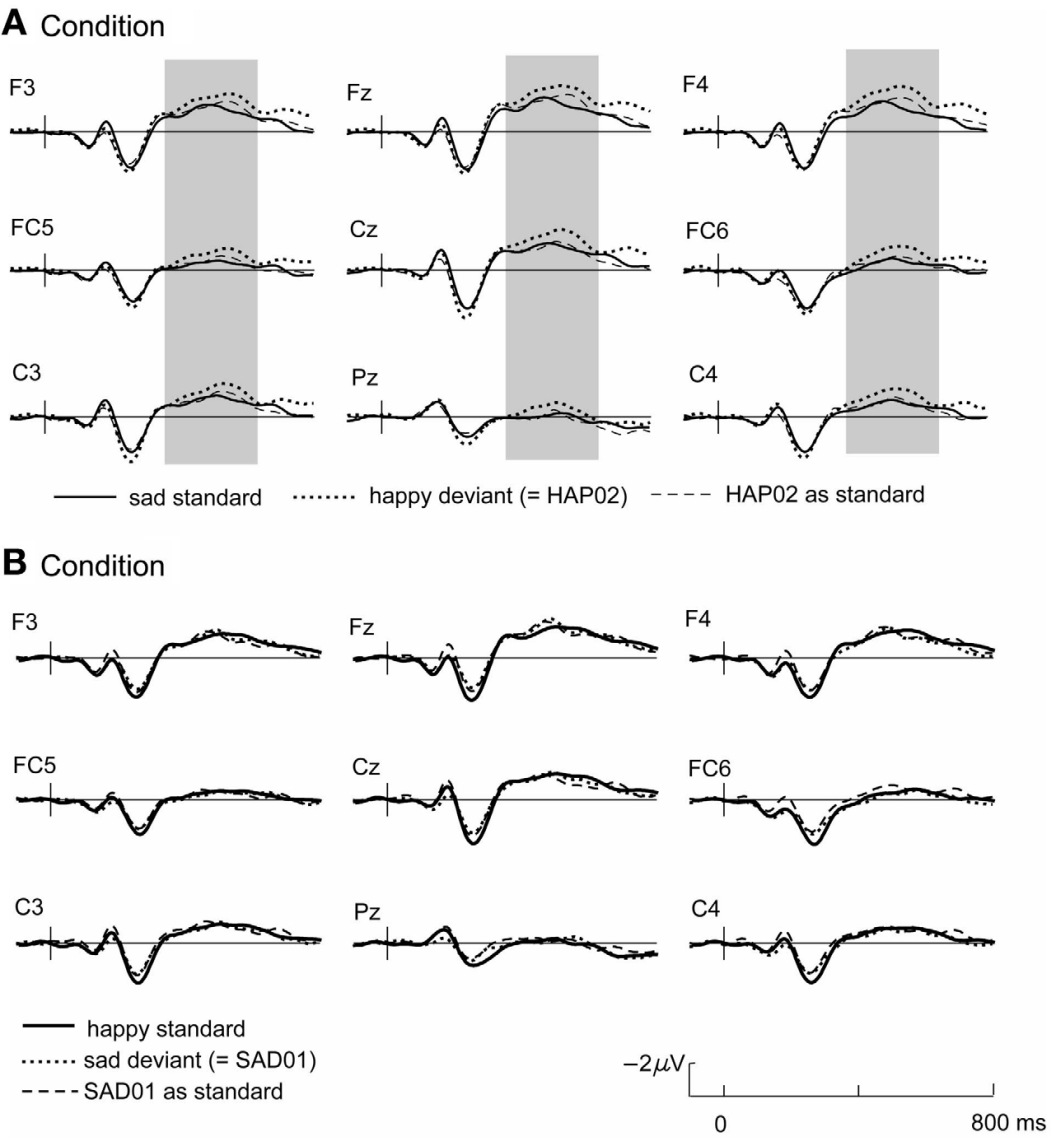
**Grand average ERPs for condition (A) (top) and (B) (bottom); the respective standard-ERP (bold line) is depicted with the ERP to the emotionally deviating tone when it was presented as deviant (dotted line) or as standard in the other condition (dashed line)**. Highlighted time windows mark significant differences in both standard-deviant comparisons.

The grand average waveforms to the standard and deviant tones (Figure 1) are characterized by a N1-P2-complex as typically found in auditory stimulation (Näätänen et al., 1988), followed by a long-duration negative component with a frontal maximum and a peak around 400–500 ms. The current design allows two different ways to assess emotional deviants. Firstly, deviants and standards collected in the same experimental blocks can be compared (i.e., happy standard vs. sad deviant or sad standard vs. happy deviant). These stimulus classes are emotionally as well as physically different. Secondly, the ERP to the deviant can be compared with the same tone when it was presented as standard in the other condition, such that the compared stimuli are physically identical but differ in their functional significance as standard and deviant (i.e., sad standard vs. sad deviant and happy standard vs. happy deviant, see Table 5). Time windows for the statistical analysis were set as follows: 100–200 ms (N1), 200–300 ms (P2), and 380–600 ms. Electrode sites included in the analysis were F3, F4, FC5, FC6, C3, C4, Fz, FCz, Cz.

**Table 5:** **Comparison of standard vs. deviant stimuli**.

**Comparison**	**Standard**	**Deviant**	**100–200 ms**	**200–300 ms**	**380–600 ms**
Condition A	Sad standards	HAP02	0.93	2.40	7.32^*^
Condition B	Happy standards	SAD01	0.06	10.94^**^	0.00
Across conditions	HAP02 as std.	HAP02	0.27	0.55	9.20^**^
Across conditions	SAD01 as std.	SAD01	3.04	0.00	0.01

Given are the F-values (df = 1,15).

** p < 0.01;

* p< 0.05.

**Figure 2:**
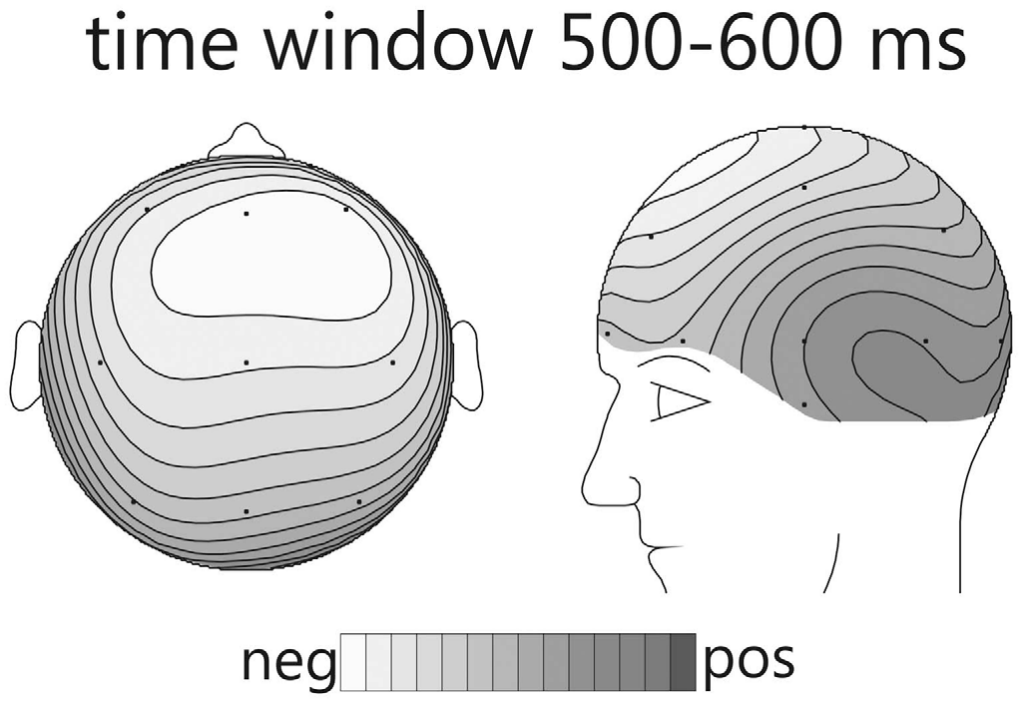
**Spline-interpolated isovoltage maps depicting the mean amplitude of the “happy deviant minus sad standard” difference wave from condition A**. A typical frontal maximum was observed. The polarity inversion at temporobasal electrodes suggests that this response belongs to the MMN family.

In condition A, emotional (happy) deviants elicited a more negative waveform in a late latency range (from 380 ms), regardless of the comparison (Figure 1, top; Table 5). Thus, the mismatch response cannot be explained by the fact that physically different tones elicited the different ERP waveforms. To illustrate the scalp distribution of this effect, the difference happy deviant minus sad standards was computed and the mean amplitude of the difference waveform in the time-window 500–600 ms was used to create spline-interpolated isovoltage maps. The topographical distribution was typical for an MMN response. In particular, we observed a polarity inversion at temporobasal (mastoid) electrode sites (Figure 2). In condition B (Figure 1, bottom; Table 5), sad deviants, too, elicited a more negative waveform than the happy standards, though in an earlier latency range (P2, 200–300 ms). However, no difference was found when the ERPs to the sad tone were compared across conditions, suggesting that this effect was triggered by the structural difference of happy and sad tones rather than their functional significance as standard and deviant. To summarize the result: presenting a happy tone in a series of sad tones resulted in a late negativity that was larger in amplitude than the ERP to the same happy tone functioning as standard in the opposite condition. In contrast, no difference that could be related to its functional significance was found for the sad tone presented in a train of differing happy tones.

## Discussion

The affective deviant in condition A evoked a clear mismatch reaction. Though the latency was rather long, its topographic distribution, including the typical inversion of polarity over temporal regions (see Figure 2) in our nose-tip referenced data, suggests that it belongs to the MMN-family. Indeed, it is a known fact that MMN-latency increases with discrimination difficulty. In this regard, we would like to point to the predecessor study (Goydke et al., 2004), in which we obtained a rather long latency of the MMN response for emotional deviants, even though the latency was still shorter than in the present study. No doubt, discrimination was particularly difficult in the present experiment, because the difference in timbre was reduced to subtle changes in the expression of same-pitch and same-instrument tones. The mismatch reaction observed for condition A suggests that a happy tone was pre-attentively categorized as different from a group of different sad tones. An MMN reflects change detection in a previously established context (Näätänen, 1992). Thus, for it to occur, a context needs to be set up first. Consequently, the important question in the present experiment is not, what is so particular about the happy tone? The question is, what has led to grouping the standard (sad) tones into one mutual category, so that the single happy tone was perceived as standing out? For the happy tone to be categorized as deviant it was required that the sad tones—though different in structure—were perceived as belonging to the same context, i.e., category. The question thus, arises: what has led to grouping of the sad tones? Three possibilities seem plausible:

- perceptual similarity
- emotional similarity or
- emotion-specific perceptual similarity

### Perceptual similarity

**Figure 3:**
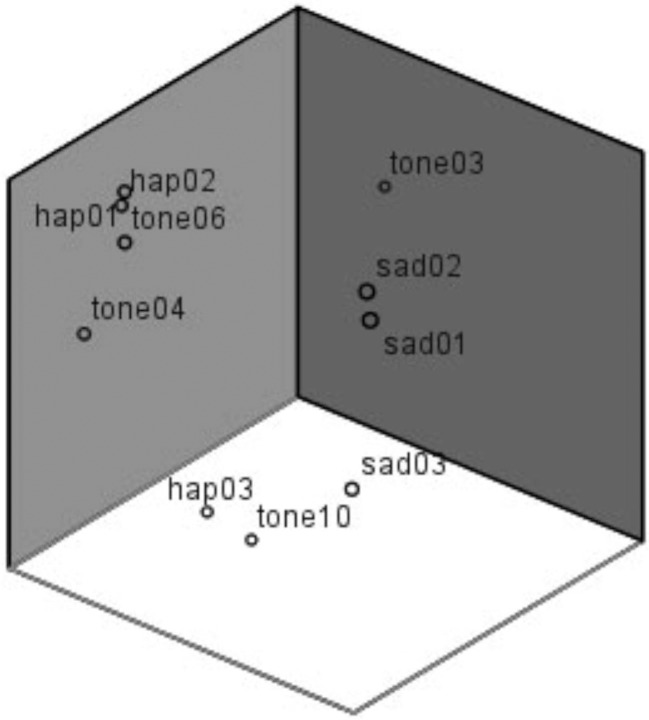
**Arrangement of tones in a three dimensional space based on the multidimensional scaling procedure**. Note that orientation of dimensions is arbitrary.

From the result of the scaling-experiment it can be derived, that tones within the sad category were perceived quite as different from each other on a perceptual basis (e.g., sad01 and sad03: Fechnerian distance = 1.29) as was the happy deviant from the sad standards (e.g., sad03 vs. happy deviant: Fechnerian distance = 1.44). Relative distances are visualized in Figure 3. The arrangement of tones in a three dimensional space results from feeding Fechnerian distance values into a MDS procedure (Alscal in SPSS) which finds the optimal constellation of stimuli in an *n*-dimensional space based on dissimilarity data. Three dimensions were found to explain 99% of variance. Note that the orientation of the dimensions is arbitrary. Though the positions of SAD01 and SAD02 are relatively close, both are rather distant from SAD03. Grouping, thus, cannot be explained by perceptual similarity alone.

### Emotional similarity

**Figure 4:**
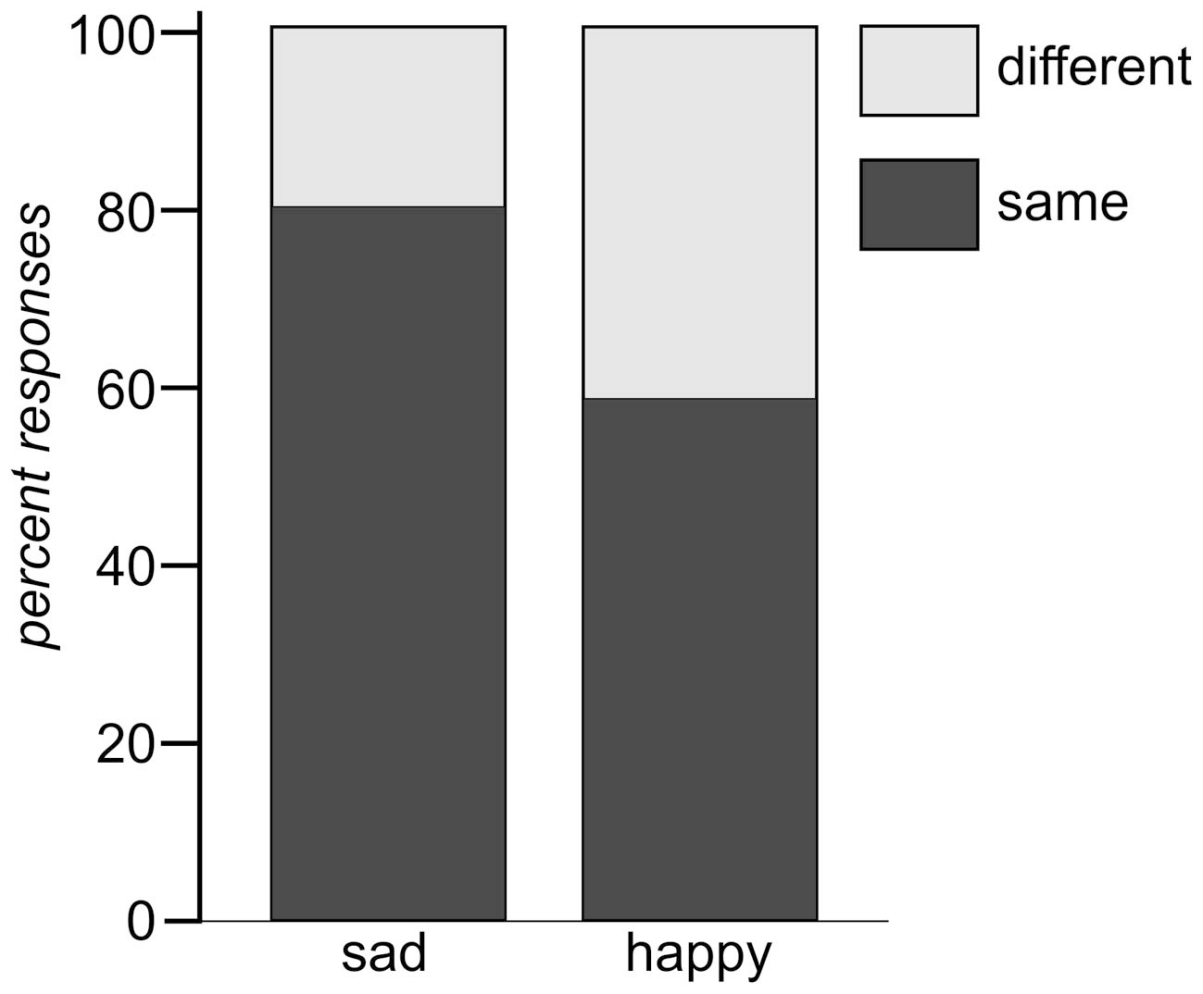
**Same and different responses for tone pairs in the categories sad (left) and happy (right), respectively**.

Affect ratings (1.90, 1.95, and 2.20) indicate that the tones were perceived as equally sad in expression. There thus, is some support for the hypothesis that the tones were grouped together based on their emotional category. However, if it was the emotional expression that has led to the automatic categorization why did it not work in condition B? No index was found for a mismatch reaction in response to a sad tone randomly interspersed in a train of different happy tones. Arguing along the same line as before, this (non)-finding implies that either no mutual standard memory trace was built for the happy tones or that this memory trace was considerably weaker for these tones. Since the affect ratings of the happy tones were just as homogeneous (3.35, 3.45, and 3.60) as those of the sad tones, the question arises, if the affect ratings gave a good enough representation of the emotion as it was decoded by the listeners. Against the background that decoding accuracy of acoustical emotion expressions has repeatedly been reported to be better for sadness than for happiness (Johnstone and Scherer, 2000; Elfenbein and Ambady, 2002; Juslin and Laukka, 2003), it might be necessary to take a second look at the stimulus material. Banse and Scherer (1996) found that if participants had the option to choose among many different emotional labels to rate an example of vocal expression, happiness was often confused with other emotions. In the present experiment participants had given their rating on bipolar dimensions ranging from happy to sad. It cannot be ruled out that the response format biased the outcome. It is, for example, possible that in some cases participants chose to rate happy because the tone was found to be definitely not-sad, even if it was not perceived as being really happy either. In an attempt to examine the perceived similarity of the tones with respect to the expressed emotion without pre-selected response categories, a similarity rating on emotional expression was performed *post-hoc*. For that purpose, the same students who had participated in the first scaling-experiment were asked to perform another same-different-judgment on the same stimulus material, though this time with regard to the emotion expressed in the tone. The results are depicted in Table 6 and show that sad tones (t.01, t.02, t.05) were perceived considerably more similar to each other with respect to the emotion expressed than the happy tones (t.07, t.08, t.09). In fact, sad tones were judged half as dissimilar from each other than the happy tones (0.503 vs. 1.02). Figure 4 shows the relation of same and different responses given for happy and sad tone pairs, respectively. Sad tones were considerably more often considered to belong to the same emotional category than happy tones (80% vs. 57% “same”-responses). It can be assumed that in the MMN-experiment, too, sad tones (in condition A) were perceived as belonging into one emotional category while happy tones (in condition B) were not. The difficulty to attribute the happy tones to the same “standard” category can serve as explanation why the sad tone did not evoke a MMN. It was not registered as deviant against a happy context, because no such context existed. Nevertheless, the hypothesis that the MMN reflects deviance detection based on emotional categorization can at least be maintained for condition A.

**Table 6:** **Fechnerian distances as calculated from same-different-judgments of emotional expression for the 10 tones**.

	**tone01**	**tone02**	**tone03**	**tone04**	**tone05**	**tone06**	**tone07**	**tone08**	**tone09**	**tone10**
t.01	0.000	0.012	1.763	1.003	0.491	0.943	1.103	1.003	1.072	0.983
t.02	0.012	0.000	1.751	0.991	0.503	0.931	1.091	0.991	1.072	0.971
t.03	1.763	1.751	0.000	1.390	1.700	1.040	0.880	0.990	1.420	1.560
t.04	1.003	0.991	1.390	0.000	0.820	0.580	0.630	0.620	0.600	0.750
t.05	0.491	0.503	1.700	0.820	0.000	1.020	1.170	1.080	0.730	0.650
t.06	0.943	0.931	1.040	0.580	1.020	0.000	0.160	0.060	0.860	0.850
t.07	1.103	1.091	0.880	0.630	1.170	0.160	0.000	0.110	1.020	1.010
t.08	1.003	0.991	0.990	0.620	1.080	0.060	0.110	0.000	0.920	0.910
t.09	1.072	1.072	1.420	0.600	0.730	0.860	1.020	0.920	0.000	0.150
t.10	0.983	0.971	1.560	0.750	0.650	0.850	1.010	0.910	0.150	0.000

Given are perceived distances of row tones and column tones with respect to their emotional expression; sad tones were t.01, t.02, and t.05, happy tones were t.07, t.08, and t.09.

### Emotion-specific perceptual similarity

It was presupposed that emotion recognition in acoustical stimuli is based on certain acoustical cues coding the emotion intended to be expressed by the sender. To test whether the sad tones in the present experiment were similar with regard to prototypical cues for sadness an acoustical analysis was performed on the stimulus set. Tones were analyzed on the parameters found to be relevant in the expression of emotion on single tones (Juslin, 2001). Using PRAAT (Boersma, 2001) and dBSonic, tones were assessed for the following features: high frequency energy, attack, mean pitch, pitch contour, vibrato amplitude, vibrato rate, sound level. For each feature, the range of values was divided into three categories (low, medium, high) and each tone was classified accordingly (Table 7). The acoustical analysis revealed that some though not all parameters were manipulated the way it would have been expected based on previous findings. However, Table 7 indicates that the cues were not used homogeneously. For example, mean pitch level was not a reliable cue. Moreover, vibrato was manipulated in individual ways by the musicians. Timbre, however, was well in line with expectations. All sad tones were characterized by little energy in the high frequency spectrum. In contrast, more energy in high frequencies was found in the spectrum of the deviant happy tone. Based on the findings by Tervaniemi et al. (1994) it appears that a difference in spectral structure alone can trigger the MMN. That would mean that the sad tones were grouped together as standards based on their mutual feature of attenuated higher partials. It has to be noted though that the high-frequency energy parameter is a very coarse means to describe timbre. Especially in natural tones [compared to synthesized tones as used by Tervaniemi et al. (1994)] the spectrum comprises a large number of frequencies with different relative intensities. As a consequence, the tones still have very individual spectra (and consequently sounds), even if they all display a relatively low high-frequency energy level. This fact is also reflected in the low perceptual similarity ratings. Moreover, if the spectral structure really was the major grouping principle, it should also have applied to the happy tones in condition B. Here, all happy tones were characterized by a high amount of energy in high frequencies, while the sad deviant was not. Nevertheless, no MMN was triggered. To conclude, though the possibility cannot be completely ruled out, it is not very likely that the grouping of the sad tones was based solely on similarities of timbre structure. Instead, the heterogeneity of parameters in Table 7 provides support for Juslin's idea of redundant code usage in emotion communication (Juslin, 1997b, 2001). Obviously, expressive cues were combined differently in different sad tones. Thus, though the sad tones did not display homogeneous patterns of emotion-specific cues, each tone was characterized by at least two prototypical cues for sadness expression. Based on the model assumption of redundant code usage, it seems likely that tones were grouped together because they were identified as belonging to one emotional category based on emotion specific-cues.

**Table 7:** **Results of the acoustical analysis of the sad tones**.

	**SAD01**	**SAD02**	**SAD03**
Timbre (high frequency energy)	Low	Low	Low
Attack	Medium	Medium	Medium
Mean pitch	Low	Medium	Medium
Pitch contour	Normal	Down	Down
Vibrato amplitude	Medium	Medium	Low
Vibrato rate	Slow	Medium	Slow
Sound level	Low	Medium	Medium

Tested were parameters expected to be relevant cues to express emotion on single tones. Categorization as low, medium, and high was based on comparison with the “happy” tones.

What implication does this consideration have for the question of grouping principles in the MMN-experiment? From what is known about the principles of the MMN, the results imply that the representation of the standard in memory included invariances across several different physical features. The invariances, however, needed to be in line with a certain template on how sadness is acoustically encoded. Several researchers have suggested the existence of such hard-wired templates for the rapid processing of emotional signals (Lazarus, 1991; LeDoux, 1991; Ekman, 1999; Scherer, 2001). It is assumed that to allow for quick adaptational behavior, stimulus evaluation happens fast and automatic. Incoming stimuli are expected to run through a matching process in which comparison with a number of schemes or templates takes place. Templates can be innate and/or formed by social learning (Ekman, 1999). The present study, while blind with respect to the origin of the template, provides some information as to how such a matching process might be performed on a pre-attentive level. Given the long latency of the MMN in the present experiment, it can be assumed that basic sensory processing has already taken place before the mismatch reaction occurs. Therefore, the MMN in the current experiment appears to reflect the mismatch between the pattern of acoustic cues identified as emotionally significant and the template for sad stimuli activated by the preceding standard tones. Our data is thus, in line with considerations that the MMN does not only occur in response to basic acoustical feature processing. Several authors have suggested that the MMN can also reflect “holistic” (Gomes et al., 1997; Sussman et al., 1998) or “gestalt-like” (Lattner et al., 2005) perception. They assume that the representation of the “standard” in the auditory memory system is not merely built up based on the just presented standard-stimuli, but that it can be influenced by prototypical representations stored in other areas of the brain (Phillips et al., 2000). Evidence from a speech-specific phoneme processing task suggested that the MMN-response does not only rely on matching processes in the transient memory store but that long-term representations for prototypical stimuli were accessed already at a pre-attentive level. For phonemes, (Näätänen and Winkler, 1999) assumed the existence of long-term memory traces serving as recognition patterns or templates in speech perception. He further posited that these can be activated by sounds “nearly matching with the phoneme-specific invariant codes” (p. 14). In another contribution, Näätänen et al. (2005) point out that the “mechanisms of generation of these more cognitive kinds of MMNs of course involve other, obviously higher-order, neural populations than those activated by a mere frequency change.” (p. 27).

In the model of Schirmer and Kotz (2006) emotional-prosodic processing is conceptualized as a hierarchical process. Stage 1 comprises initial sensory processing of the auditory information before emotionally significant cues are integrated (stage 2) and cognitive evaluation processes (stage 3) take place. The MMN in response to emotional auditory stimuli might reflect the stage of integrating emotionally significant cues (Schirmer et al., 2005). The present data is compatible with the model albeit in the area of nonverbal auditory emotion processing. The current data contributes to disentangling the processes underlying emotion recognition in the auditory domain. It has to be pointed out though that the present results can only give a first glimpse on the mechanisms underlying processing of emotionally expressive tones. More studies with a larger set of tones characterized by different cues are needed to systematically examine the nature of the stimulus evaluation process.

Also, a critical issue for emotion recognition from musical sounds might be the time over which a listener can integrate the information. This might be the answer to the question as to why the happy tones were perceived less homogeneous than the sad tones. While all musicians had the intention to express happiness, it is possible that happiness can just not be expressed very well on single tones. Juslin (1997a), when looking for predictors of emotional ratings of musical performances, found that the best predictors for happiness were tempo and articulation. Both parameters are suprasegmental features and require a whole sequence of tones. In contrast, sadness ratings could be predicted by a number of cues, including segmental features such as sound level, spectrum, and attack.

### Conflict of interest statement

The authors declare that the research was conducted in the absence of any commercial or financial relationships that could be construed as a potential conflict of interest.
